# Surgical treatment of nasal packing refractory epistaxis

**DOI:** 10.1016/S1808-8694(15)30647-9

**Published:** 2015-10-19

**Authors:** Fábio Augusto Winckler Rabelo, Vírgilio Batista do Prado, Fabiana Cardoso Pereira Valera, Ricardo Cassiano Demarco, Edwin Tamashiro, Wilma Terezinha Anselmo Lima

**Affiliations:** 1 MD, Resident Physician; 2 MD, Resident Physician; 3 PhD, Assistant Physician; 4 MSc, Assistant Physician; 5 MD, PhD student; 6 Associate Professor. Faculdade de Medicina de Ribeirão Preto - Universidade de São Paulo

**Keywords:** cautery, epistaxis, ligation

## Abstract

Epistaxis is the main otorhinolaryngology emergency and, in severe cases, it can lead to hemodynamic instability and be life threatening.

**Aim:**

To evaluate factors involved in epistaxis resistant to nasal packing that needed surgical treatment, as well as post-surgical results.

**Materials and Methods:**

Retrospective study from January 2002 to August 2007. 40 consecutive patients that underwent surgical treatment for refractory epistaxis were analyzed. Predisposing factors, procedures performed, need of blood transfusion, and recurrence were evaluated.

**Results:**

Otorhinolaryngology post operative complications (37.5%), high blood pressure (30%), and coagulopathy (15%) were the main factors related to epistaxis. 50% of the patients (n=20) presented with hemodynamic instability and 90% of them (n=18) needed blood transfusion. Eletrocauterization of the bleeding site was enough in 35% of these patients (n=14), while in 65% (n=26) was necessary cauterization and/or arterial ligation. Five patients (12.5%) had bleeding recurrence, which needed re-operation.

**Conclusion:**

Earlier indications of surgical treatment to control severe and refractory epistaxis to conventional treatment, especially in a population with high risk such as post operative bleeding and coagulopathies, may decrease the need of blood transfusion.

## INTRODUCTION

Epistaxis is a quite common finding in our daily practice, and is considered to be the main emergency in the ENT realm. Studies have shown that up to 60% of the population is affected by epistaxis at some point in their lives^1^,^2^. In most cases bleedings are small and self-limited, but the few severe ones may adversely affect the patient's hemodynamic stability and put his/her life at risk.

The causes of epistaxis may be either local or systemic. Bleeding location, severity, and patient evolution are usually determining factors in guiding therapy. In cases of severe epistaxis, where nasal packing fails to contain the bleeding or bleeding relapses after packing removal or the patient cannot tolerate or refuses to be placed with a nasal pack, surgery^3^ is required. Surgical procedure options range between electrical or chemical cauterization of localized bleeding sites and/or ligation of the anterior and posterior ethmoidal arteries or branches of the sphenopalatine artery when bleeding is more profuse and diffuse.

In order to better characterize this patient population, assess their risk levels, and evaluate proposed surgical treatment effectiveness, we decided to look into individual conditions connected to refractoriness to conventional epistaxis care and postoperative outcome.

## OBJECTIVES

This paper aims to analyze clinical aspects and factors connected to etiology, as well as cauterization and/or vascular ligation effectiveness in epistaxis cases refractory to conventional treatment.

## MATERIALS AND METHOD

This study was approved by the Research Ethics Committee at Hospital das Clínicas da Faculdade de Medicina de Ribeirão Preto under permit 12922/2006. This is a retrospective cross-sectional study in which 40 consecutive patients requiring surgery for epistaxis between January of 2002 and August of 2007 were enrolled. Patients were assessed at the ENT Service of the Hospital das Clínicas de Ribeirão Preto - Universidade de São Paulo. They were first treated with anterior and/or posterior nasal packing to manage epistaxis, as dictated by the study's design. No patients were excluded.

During their stay at the university hospital, patients were tested for hemoglobin levels, packed red cell volume, platelet count, prothrombin time (PT/INR), activated partial thromboplastin time (APTT), liver function in cases suspected with liver disease, and vital signs. All surgical procedures were conducted under general anesthesia and endoscopic visualization with 0 and 45° scopes of 4mm in diameter. Patients were also analyzed in terms of gender, age, presence of comorbidities such as systemic arterial hypertension, liver disease, blood dyscrasia, need for blood transfusion, conducted surgical procedure, relapse, and other postoperative complications.

Electrical cauterization was the procedure of choice for situations in which bleeding was punctual and subject to accurate location. When the site of bleeding could not be precisely located or hemorrhage was more profuse, artery ligation was performed according to bleeding topography. Sphenopalatine artery branch ligation procedures also included the lifting of a mucoperiosteal flap 1cm anteriorly and inferiorly to the most caudal portion of the middle turbinate, until the site of the sphenopalatine foramen close to the ethmoidal crest. When the arterial branches were identified and exposed, the artery was clipped and cauterized with a bipolar forceps. Cases in which the site of bleeding was located more superiorly, we chose to ligate the anterior ethmoidal arteries via Lynch's external incision.

## RESULTS

### Patients

Forty patients – 27 (67.5%) females and 13 (32.5%) females – were enrolled in the study. Ages ranged between 4 and 78 years (40 ± 20 years, median ± SD).

### Predisposing Factors /History

**Figure 1:**
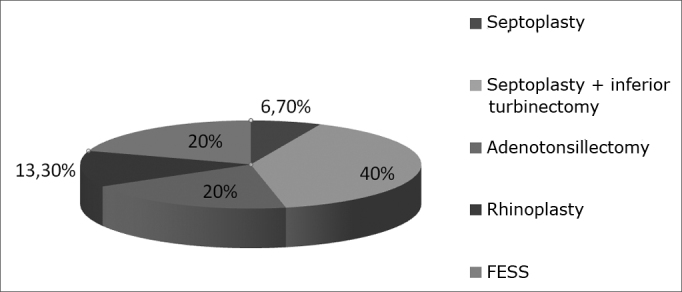
Distribution of epistaxis related to postoperative procedures

In 15 patients (37.5%) epistaxis appeared as a postoperative complication in ENT surgical procedures (septoplasty combined or not with turbinectomy, adenotonsillectomy, rhinoplasty, or paranasal sinus endoscopic surgery) (Fig. 1). Eight patients (53%) had immediate postoperative bleeding (<48 hours) and all others had bleeding episodes after the first 48 hours. Only one patient had history of recent external nose trauma (2.5%).

**Figure 2:**
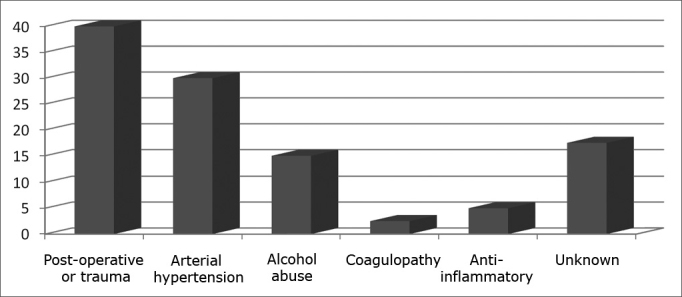
Prevalence of factors associated with epistaxis in patients with epistaxis refractory to conventional treatment.

Recent use of anticoagulants or non-hormonal anti-inflammatory drugs was observed in two patients. Six patients (15%) had history of alcohol abuse, but none of them presented altered liver function or coagulation test results. Only one patient had a coagulation disorder (Glanzmann's thrombasthenia). Twelve patients (30%) had previous history of multiple episodes of nasal bleeding. Seven patients (17.5%) had no evident predisposing factor for epistaxis (Fig. 2).

### Surgical Procedure

Sixteen (40%) of the 40 operated patients required bilateral surgical approach, while the remaining 24 (60%) were approached unilaterally.

Cauterization of the bleeding site was performed in 14 (35%) patients; 11 of them were treated postoperatively after nasal surgery. The most frequently cauterized sites were the inferior turbinates (n=6), the rhinopharynx (n=3) and Little's area (n=2). In this group of patients, 12 (86%) of the 14 cases were successfully treated. One of the relapsing patients was treated with review surgery, while the other was offered anterior packing (Table 1).

**Table 1:** Assessment and conduct in immediate postoperative relapses.

Patient	Offered procedure	Procedure after relapse
2	Clip anterior ethmoidal and contralateral posterior lateral nasal arteries	Bilateral anterior nasal packing
9	Clip right posterior lateral nasal artery	Clip right anterior ethmoidal artery
10	Clip right posterior lateral nasal artery	Clip right anterior ethmoidal artery
21	Left inferior turbinate tail cauterization	Clip left posterior lateral nasal artery
35	Septum cauterization	Bilateral anterior nasal packing

Sphenopalatine artery branch cauterization was performed in 26 (65%) patients; 18 were treated unilaterally and 8 bilaterally. Anterior ethmoidal artery ligation was performed alone in five (12.5%) patients and combined with sphenopalatine artery branch in 10 (25%) patients. Three (11.6%) patients treated with arterial ligation relapsed. Surgical review revealed incomplete ligation with remaining terminal branches or presence of bleeding in the topography of other vascular beds (Table 1).

No patients had significant complaints or complications related to either the cauterization or vascular ligation procedures.

### Hemodynamic Instability

Hemoglobin level variation found in the patients between the time they arrived at our service and entered surgery was −2.24 g/l (11.8±1.9 × 8.9±2.6; median ± SD). Some cases were quite severe, and 20 (50%) patients were hemodinamycally instable. Nineteen of were given blood transfusions with packed red blood cells. One was given volume expanders for religious reasons.

## DISCUSSION

According to the literature, local trauma is the main cause of epistaxis^4^,^5^. However, 37.5% of the cases of epistaxis were connected to postoperative complications of ENT surgical procedures. Such high prevalence of postoperative cases is firstly due to the fact that most external or multiple trauma patients are not referred to or treated at our ENT service, but by the trauma teams. That explains the proportional increase in the prevalence rates of epistaxis connected to other causes. Furthermore, ours is a tertiary care center strongly focused on surgery and equipped with a training service for resident physicians. Most of the observed cases of postoperative epistaxis occurred after inferior partial turbinectomy (40%), possibly due to incomplete cauterization of the remaining inferior nasal concha bed.

Systemic hypertension has been broadly discussed as a cause of epistaxis. Recent studies have shown little correlation between systemic hypertension and epistaxis^6^, ^7^, ^8^, ^9^. Although 30% of our patients had positive history of systemic hypertension, most of them did not have high blood pressure levels during bleeding that could account for the severity of their epistaxis episodes.

Alcohol abuse is generally associated with poor nutrition and vitamin C and K deficiency. Healing processes and coagulation factor production (factors II, V, VII and IX and fibrinogen and prothrombin more specifically) are consequently impaired, thus increasing the incidence of bleeding episodes. Signs of liver failure were however not found in our patients, not even in the ones with a history of alcohol abuse.

Use of drugs that affect coagulation (acetylsalicylic acid, anticoagulants and non-hormonal anti-inflammatory drugs) must be investigated in the etiology of epistaxis and considered in the rendering of a differential diagnosis. Only a small portion of the patients in our group (10%) had epistaxis associated with the use of this category of drugs.

One patient had Glanzmann's thrombasthenia, a rare recessive hemorrhagic syndrome^10^ that introduces a quantitative and/or qualitative deficit on a platelet membrane glycoprotein complex thus reducing platelet adhesion and aggregation. We chose to perform a bilateral arterial ligation in this patient, as the child required frequent red blood cell and platelet transfusions due to frequent and profuse bleeding episodes. A significant reduction on both the frequency and severity of the bleeding episodes was observed after surgery.

In our practice we try to guide the management of epistaxis by the topography of the bleeding site. When a punctual focus of hemorrhage can be pinpointed, a simple electrical cauterization is performed, as seen mainly in the immediate postoperative epistaxis events. When bleeding is more profuse and diffuse, and the site of bleeding cannot be located, the nasal cavity terminal irrigation arteries (such as the branches of the sphenopalatine and anterior ethmoidal arteries) are ligated. The choice between clipping and cauterization is based on the surgeon's familiarity with the procedure and the visualization of the branches to be occluded. There is no clear evidence in the literature to state that either is more effective^11^. Our results show that in 88.4% of the cases epistaxis was completely managed after arterial ligation. The two cases in which epistaxis relapsed after sphenopalatine artery ligation were ultimately resolved after a review procedure was performed and the remaining terminal branches of the artery and the anterior ethmoidal artery were ligated.

Relapses after arterial ligation are infrequent, but may occur due to a few reasons: failure to identify the site of bleeding, failure to identify the sphenopalatine foramen, failure to ligate all the branches of the sphenopalatine artery, and coagulation disorders^12^,^13^.

Patients in our group submitted to surgery for epistaxis already presented reduced serum hemoglobin levels at their admission, and further reductions were observed as they were offered the conventional treatment, which proved to be ineffective. Barlow et al.^14^ have shown a strong association between surgery for epistaxis and need for blood transfusion. Likewise, Voegels et al.^15^ reported similar rates of blood transfusion (45.5%) in patients submitted to sphenopalatine artery ligation. These findings support the idea that, in severe cases and patients at risk for hemodynamic instability, earlier surgery could reduce the need for blood transfusion. None of the patients had complaints or complications connected to the surgical procedure done to manage their epistaxis, proving the safety of the method.

## CONCLUSION

Surgery to manage epistaxis refractory to nasal packing is highly effective, safe, and presents low complication rates. When offered to patients with predisposing factors for bleeding and at high risk of hemodynamic instability, early surgery can reduce the need for blood transfusion.
